# Dispositional optimism and all-cause mortality after esophageal cancer surgery: a nationwide population-based cohort study

**DOI:** 10.1007/s00520-022-07311-z

**Published:** 2022-08-11

**Authors:** Yangjun Liu, Erik Pettersson, Anna Schandl, Sheraz Markar, Asif Johar, Pernilla Lagergren

**Affiliations:** 1 Department of Molecular Medicine and Surgery, Karolinska Institutet, Karolinska University Hospital, Retzius väg 13a, Level 4, 171 77 Stockholm, Sweden; 2 Department of Medical Epidemiology and Biostatistics, Karolinska Institutet, Stockholm, Sweden; 3 Department of Anaesthesiology and Intensive Care, Södersjukhuset, Stockholm, Sweden; 4 Department of Clinical Science and Education, Södersjukhuset, Karolinska Institutet, Stockholm, Sweden; 5 Nuffield Department of Surgery, University of Oxford, Oxford, UK; 6 Department of Surgery and Cancer, Imperial College London, London, UK

**Keywords:** Survival, Survivorship, Positive psychology, Esophagectomy, COVID-19

## Abstract

**Purpose:**

To examine the association between dispositional optimism and all-cause mortality after esophageal cancer surgery and whether pathological tumor stage and the COVID-19 pandemic modified this association.

**Methods:**

This nationwide, population-based prospective cohort study included 335 patients undergoing esophageal cancer surgery in Sweden between January 1, 2013, and December 31, 2019. Dispositional optimism was measured 1 year post-surgery using Life Orientation Test-Revised (LOT-R). A higher LOT-R sum score represents higher dispositional optimism. Mortality information was obtained from the Swedish Register of the Total Population. All patients were followed up until death or until December 31, 2020, whichever occurred first. Cox regression with adjustments for confounders was used.

**Results:**

The median follow-up was 20.8 months, during which 125 (37.3%) patients died. Among the included 335 patients, 219 (65.4%) patients had tumor pathologically staged Tis-II, and 300 (89.6%) patients entered the cohort before the COVID-19 pandemic. Both tumor stage and the COVID-19 pandemic were effect modifiers. For each unit increase in LOT-R sum score, the risk of all-cause mortality decreased by 11% (HR 0.89, 95% CI 0.81 to 0.98) among patients with tumor staged Tis-II before the COVID-19 pandemic. This association was non-significant in patients with tumor staged III–IV (HR 0.99, 95% CI 0.92 to 1.07) and during the COVID-19 pandemic (HR 1.08, 95% CI 0.94 to 1.25).

**Conclusion:**

Assessing dispositional optimism may help predict postoperative survival, especially for patients with early and intermediate esophageal cancer. Increasing dispositional optimism might be a potential intervention target to improve survival after esophageal cancer surgery.

**Supplementary Information:**

The online version contains supplementary material available at 10.1007/s00520-022-07311-z.

## Background

Esophageal cancer is the seventh most commonly diagnosed cancer worldwide [1], which carries a poor prognosis with an overall 5-year survival below 20% [2, 3]. Surgical resection (esophagectomy) is the mainstay of curative treatment. However, the 5-year survival after esophagectomy is still less than 50% as reported by a Swedish nationwide cohort study [2]. Identified prognostic factors for survival after esophageal cancer surgery include sociodemographic, clinical, and surgeon-related factors such as age, tumor stage, and surgeon volume [4–9]. However, these predictors cannot fully explain the variation of postoperative survival, and most of them are not modifiable after surgery. Thus, identifying other potentially modifiable predictors is important.

Dispositional optimism is a personality trait defined as generalized positive expectations for the future [10]. It is relatively stable but can be increased via psychological interventions [11]. Higher dispositional optimism has been found to be associated with better physical health [12] and with lower all-cause mortality and cardiovascular death in the general and elderly populations [13–21]. However, studies conducted in patients with cancer have reported inconsistent results. Higher dispositional optimism was associated with lower mortality in patients with recurrent or metastatic cancer treated with palliative radiation, head and neck cancer, and ovarian cancer [22–24]. Contrarily, there was no association in patients with metastatic colorectal cancer, wherein the reported hazard ratio (HR) equaled 0.98 based on 429 subjects [25]. To date, it remains unclear whether such an association exists in patients with esophageal cancer.

The present study aimed to use a Swedish nationwide, population-based, prospective cohort to examine whether dispositional optimism predicted all-cause mortality after esophageal cancer surgery. In addition, given that esophageal cancer is an aggressive tumor and the COVID-19 pandemic severely affects the society and healthcare system, the effect of dispositional optimism on survival might be overridden by the effect of the strongest prognostic factor, tumor stage, and of the hazard event, the COVID-19 pandemic. Therefore, we also aimed to examine the potential effect modifications by pathological tumor stage and the COVID-19 pandemic.

## Material and methods

### Study design

Data for the present study were drawn from a prospective, ongoing, Swedish nationwide, and population-based cohort study entitled Oesophageal Surgery on Cancer patients-Adaptation and Recovery (OSCAR). Details of the OSCAR study have been described elsewhere [26, 27]. In brief, all 1-year survivors without cognitive impairment undergoing esophagectomy for cancer in Sweden from January 1, 2013, and onwards are invited (approximate response rate 66%) [26]. Follow-up data included patient-reported outcomes as well as regular assessments of vital status [26]. The OSCAR study was approved by the Regional Ethical Review Board in Stockholm, Sweden (diary number 2013/844–31/1), and informed consents were obtained from all participants before inclusion.

The present study included OSCAR study participants undergoing esophageal cancer surgery in Sweden between January 1, 2013, and December 31, 2019. Patients with a psychiatric history were excluded.

### Exposure: dispositional optimism

After consenting to participate in the OSCAR study, patients self-reported their dispositional optimism on the Swedish version of Life Orientation Test-Revised (LOT-R) [28, 29] during the first interview (i.e., 1 year after esophageal cancer surgery). LOT-R consists of three positively worded items and three negatively worded items [28, 29], and the extent of agreement on these six items are indicated on a five-point Likert scale ranging from 0 (“strongly disagree”) to 4 (“strongly agree”) [28, 29].

Given the inconclusive dimensionality of LOT-R [30] and the absence of psychometric research among patients with esophagectomy for cancer, we have previously conducted a series of confirmatory factor analyses and presented the detailed results elsewhere [31]. Because the first negatively worded item had bimodal response distribution, equivocal correlations with both positively and negatively worded items, and negative loading in the best-fitting model [31], which suggest that a considerable proportion of patients most likely misread it, we removed this item [31]. The final adopted model assumes one dimension (dispositional optimism) with correlated errors between the two reversed negatively worded items [31].

A sum score of the remaining five items was computed with a higher score representing higher dispositional optimism. The internal reliability estimated by McDonald’s omega was 0.49, with 95% bootstrapped confidence interval (CI) 0.31 to 0.62 [31].

### Outcome: all-cause mortality

All patients were followed from 1 year post-surgery (identified as the date of the first interview after consenting to participate in the OSCAR study) and until the date of death or December 31, 2020, whichever occurred first. Mortality information was obtained from the Swedish Register of the Total Population, which has 100% complete ascertainment of death [32].

### Covariates

Four sociodemographic variables including age at surgery (continuous), sex (female or male), education level (9-year compulsory school, upper secondary school, or higher education), and cohabitation status (non-cohabitating or cohabitating) were considered as confirmed confounders because they are associated with both dispositional optimism and mortality [4, 33]. The following seven clinical variables were considered as potential but unconfirmed confounders as they are predictors of survival [4–7] but no available evidence has suggested that they can affect dispositional optimism [34, 35]: (1) pathological tumor stage (Tis-II or III–IV), (2) Charlson Comorbidity Index (0, 1, or ≥ 2), (3) neoadjuvant therapy (yes or no), (4) histology (squamous cell carcinoma or adenocarcinoma), (5) postoperative Clavien–Dindo complication score (none, I–II, or III–IV), (6) resection margin status (R0 or non-R0), and (7) surgical approach (minimally invasive, hybrid, or open surgery).

The sociodemographic data were collected from the patient-reported questionnaires and the Swedish Longitudinal Integration Database for Health Insurance and Labor Market Studies. Clinical data were obtained from medical records, the Swedish Patient Register, and the Swedish Cancer Register.

### Statistical analysis

The overall mean of the LOT-R sum scores between patients with different characteristics were compared using *t*-test or analysis of variance. Multivariable Cox proportional hazards model was used to calculate the HR with 95% CI for the all-cause mortality with one unit increase in the LOT-R sum score. Time since surgery was used as the time scale. The four sociodemographic variables were adjusted for in all Cox models because they were confounders based on prior knowledge [4, 33]. Given that we could not decide whether clinical variables were confounders because of the absence of prior knowledge, we used data-driven forward selection, and only covariates leading to ≥ 10% change in the estimated HR could be included in the final Cox models. The estimated HR was almost unchanged after further adjusting for clinical variables (Supplementary Table S1), and thus none of them was considered as confounders in the current study. Potential effect modification was examined by adding related interaction terms into Cox models.

We treated the COVID-19 pandemic as a time-varying variable, and the period from March 1, 2020, was regarded as within the COVID-19 pandemic in Sweden. Given that most person-time in this study was before the COVID-19 pandemic, we might only have statistical power for the analysis involving period before the COVID-19 pandemic but not period within the COVID-19 pandemic. Therefore, we conducted a subgroup analysis limiting the follow-up ending date before the occurrence of the COVID-19 pandemic in Sweden (i.e., March 1, 2020) to further examine the association during the period without the COVID-19 pandemic.

In addition, to account for the potential reverse causality that approaching death might affect dispositional optimism, we excluded patients who survived less than 2 months after the dispositional optimism measurement and re-estimated all Cox models.

The assumption of proportional hazards was tested in all Cox models using Schoenfeld residuals and it was met for all analyses. All hypothesis tests were two-sided with significance level 0.05. Stata 13 (StataCorp, College Station, Texas, USA) and SAS 9.4 (SAS Institute, Cary, NC, USA) were used for the statistical analyses.

## Results

### Study participants

**Fig. 1:**
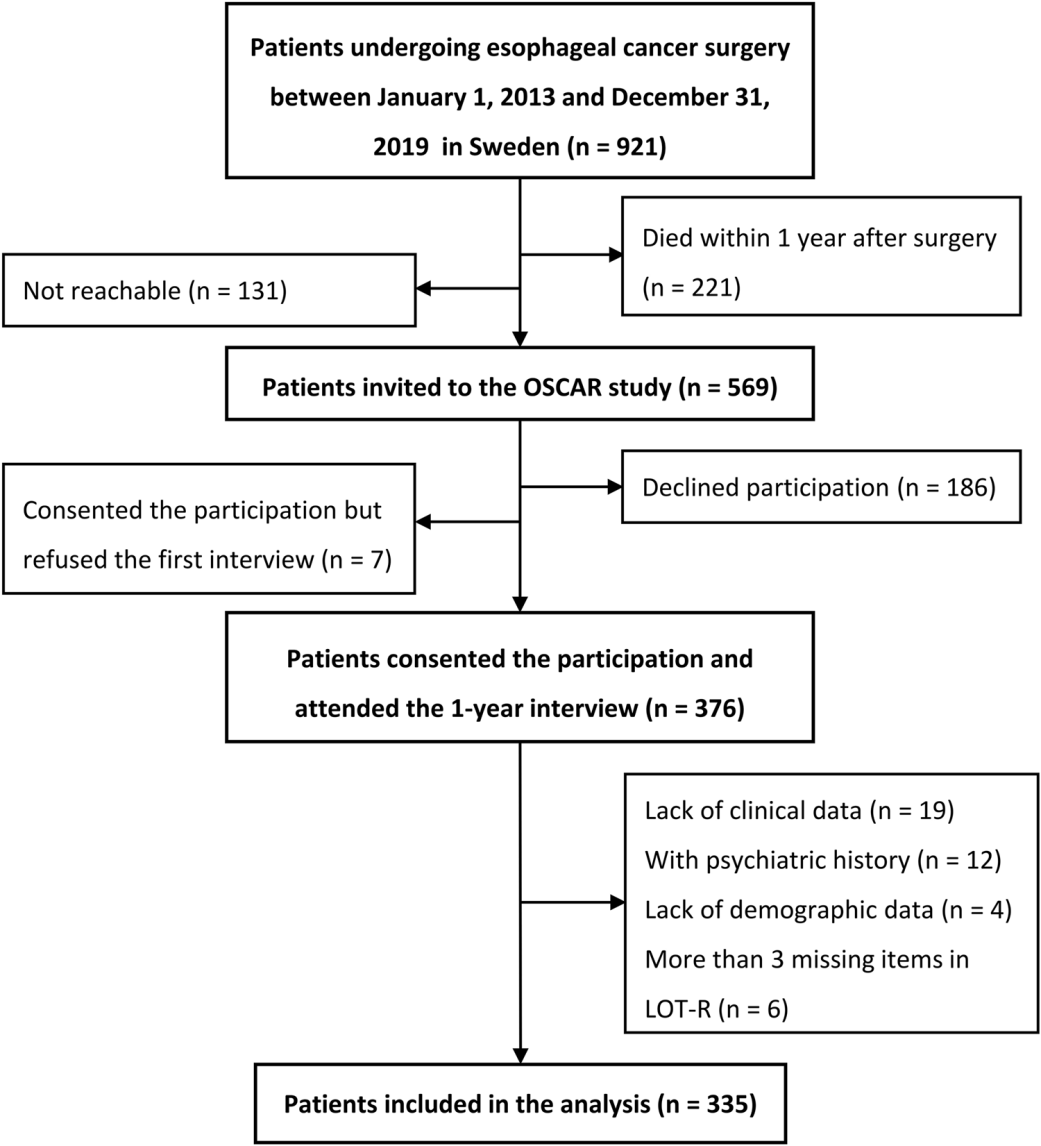
Flowchart of patient selection for inclusion. Note. OSCAR study: a prospective, ongoing, Swedish-nationwide cohort study entitled “Oesophageal Surgery on Cancer patients-Adaptation and Recovery (OSCAR).” LOT-R, Life Orientation Test-Revised

In total, 921 patients underwent esophageal cancer surgery between January 1, 2013, and December 31, 2019, in Sweden. After excluding 221 patients who died within 1 year after surgery and 131 patients who were uncontactable, 569 patients were invited to participate in the OSCAR study. Of these, 376 patients consented to the participation and accomplished the first interview. Non-participation was mainly associated with poor health, cancer recurrence, and unwillingness [26]. After further excluding 41 patients with a psychiatric history or missing data, the final study cohort included 335 patients. The detailed process of patient selection is displayed in Fig. 1.

### Characteristics of participants

Table 1 presents the characteristics of the 335 patients included in the present study. The average age at surgery was 67.4 years with a standard deviation (SD) of 8.2 years. Most patients were male (90.8%), married/cohabitating (75.4%), had pathological tumor stage Tis-II (65.4%), and entered the OSCAR study before the COVID-19 pandemic (89.6%). The mean of LOT-R sum scores was 15.2 ± 3.3 (range 5 to 20). The dispositional optimism score was comparable between patients with different characteristics (Supplementary Table S2).

**Table 1:** Characteristics of the 335 patients undergoing esophageal cancer surgery in Sweden

	**Number (%)**
**Age**	
Mean ± SD	67.4 ± 8.2
Median	68.7
Range	[34.9, 83.7]
**Sex**	
Female	31 (9.3)
Male	304 (90.8)
**Cohabitation status**	
Non-cohabitating	79 (23.6)
Cohabitating	256 (76.4)
**Education level**	
Nine-year compulsory school	82 (24.5)
Upper secondary school	159 (47.5)
Higher education	94 (28.1)
**Neoadjuvant therapy**	
Yes	265 (79.1)
No	70 (20.9)
**Surgical approach**	
Total minimally invasive esophagectomy	115 (34.3)
Hybrid minimally invasive esophagectomy	119 (35.5)
Open esophagectomy	101 (30.2)
**Pathological tumor stage**	
Tis-II	219 (65.4)
III–IV	116 (34.6)
**Tumor histology**	
Adenocarcinoma	279 (83.3)
Squamous cell carcinoma	51 (15.2)
Dysplasia	5 (1.5)
**Postoperative complications (Clavien–Dindo grade)**	
No complication	113 (33.7)
I–II	94 (28.1)
III–IV	128 (38.2)
**Resection margin status**	
Radical	307 (91.6)
Nonradical	28 (8.4)
**Charlson Comorbidity Index**	
0	142 (42.4)
1	112 (33.4)
≥ 2	81 (24.2)
**LOT-R sum score**	
Mean ± SD	15.2 ± 3.3
Median	15
Range	[5, 20]

Note. All values are number (%) unless otherwise stated, and the percentage is rounded up, which in some cases gives a sum not equal to 100%. *LOT-R*, Life Orientation Test-Revised

### All-cause mortality in the whole cohort with follow-up containing the COVID-19 pandemic

Among the 335 patients included in the whole cohort with follow-up until December 31, 2020, 125 deaths occurred. Patients were followed up for a median of 20.8 months (interquartile range 10.9 to 43.7 months) with a total of 9305.0 person-months.

**Fig. 2:**
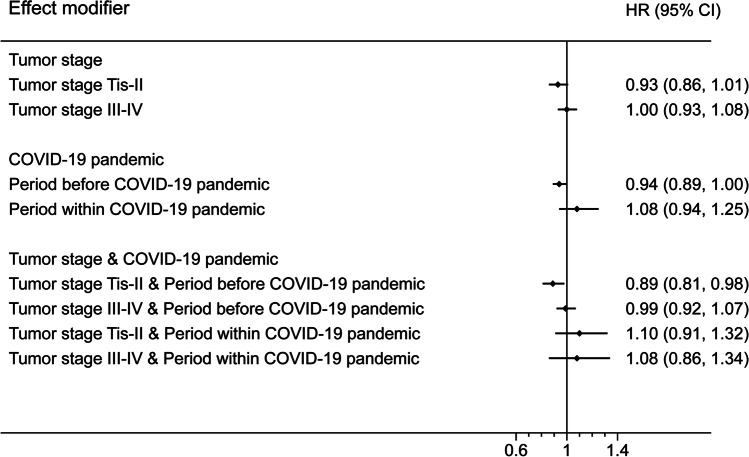
Hazard ratio (HR) and 95% confidence interval (CI) for all-cause mortality with one unit increase in the sum score of Life Orientation Test-Revised (LOT-R), with pathological tumor stage and the COVID-19 pandemic as effect modifiers

The model without any interaction terms showed that the overall HR was 0.96 (95% CI 0.91 to 1.02). The model containing an interaction term between dispositional optimism and pathological tumor stage showed that HR for patients with tumor staged Tis-II was 0.93 (95% CI 0.86 to 1.01; Fig. 2) and for patients with tumor staged III–IV was 1.00 (95% CI 0.93 to 1.08; Fig. 2). The *p*-value for the interaction term was 0.24. The model containing an interaction term between dispositional optimism and the COVID-19 pandemic showed that HR for the period before the COVID-19 pandemic was 0.94 (95% CI 0.89 to 1.00; Fig. 2) and for the period within the COVID-19 pandemic was 1.08 (95% CI 0.94 to 1.25; Fig. 2). The *p*-value for the interaction term was 0.08.

Given that the above results suggested that both pathological tumor stage and the COVID-19 pandemic were potential effect modifiers, we built a model with interaction terms among dispositional optimism, pathological tumor stage, and the COVID-19 pandemic. It showed that in the period before the COVID-19 pandemic and among patients with tumor staged Tis-II, higher dispositional optimism was significantly associated with better overall survival. For each unit increase in the LOT-R sum score, the risk of all-cause mortality decreased by 11% (HR 0.89, 95% CI 0.81 to 0.98; Fig. 2). However, during the COVID-19 pandemic and for patients with tumor staged III–IV, the associations between dispositional optimism and all-cause mortality were not statistically significant (Fig. 2).

### All-cause mortality in the sub-cohort with follow-up before the COVID-19 pandemic

In the subgroup analysis limiting the follow-up before the COVID-19 pandemic occurred in Sweden (i.e., March 1, 2020), 300 patients were included, of which 102 patients died during the follow-up with a median of 18.0 months (interquartile range 9.4 to 36.1 months) and a total of 7221.0 person-months.

**Fig. 3:**
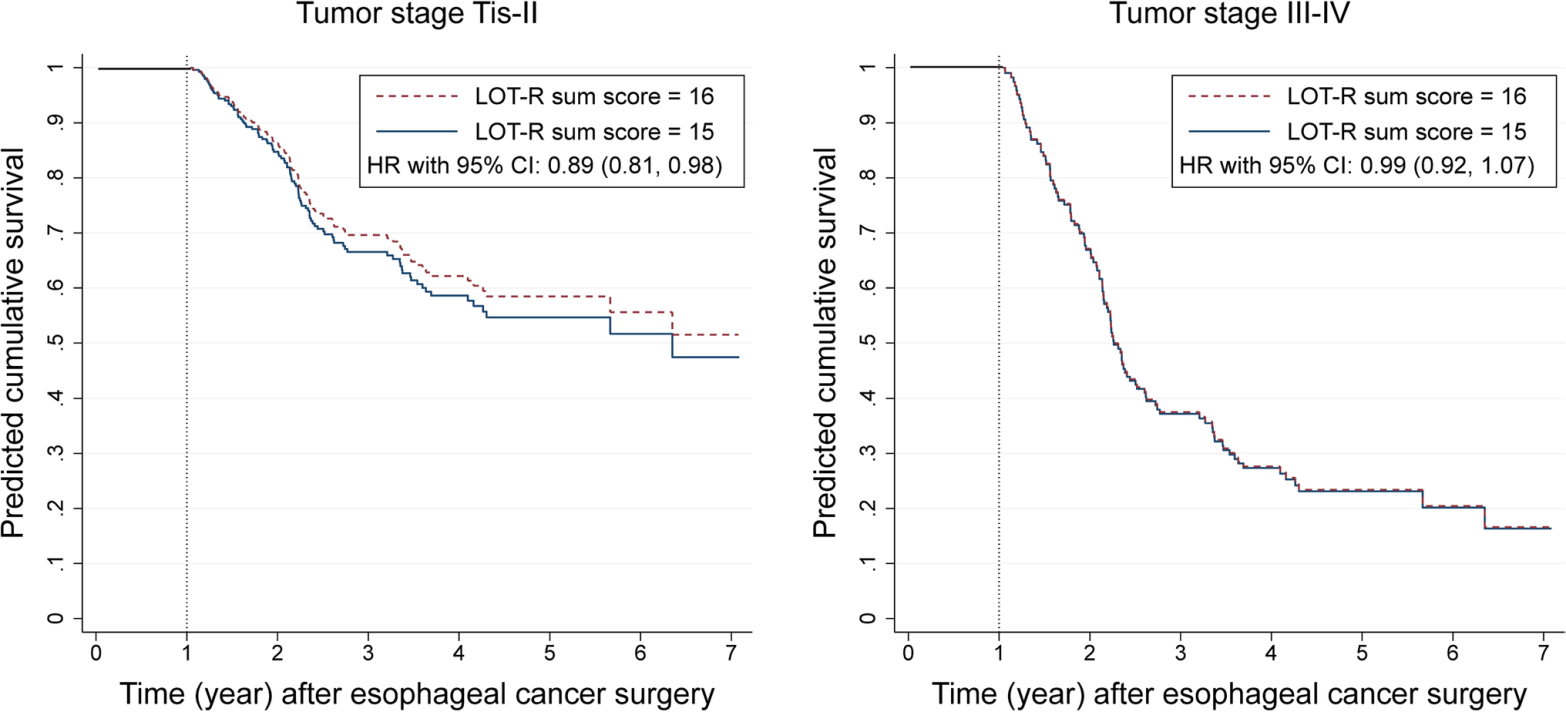
Predicted cumulative survival curves after esophageal cancer surgery for patients with average dispositional optimism level (LOT-R sum score equaling to 15) and patients with higher dispositional optimism (LOT-R sum score equaling to 16), with follow-up period before the COVID-19 pandemic and pathological tumor stage as an effect modifier. Note. LOT-R, Life Orientation Test-Revised; HR, hazard ratio; CI, confidence interval

The model without any interaction terms showed that the overall HR was 0.94 (95% CI 0.89 to 1.00). The model with an interaction term between dispositional optimism and pathological tumor stage generated similar results as the above model using the whole cohort. Higher dispositional optimism was significantly associated with lower all-cause mortality for patients with tumor staged Tis-II (HR 0.89, 95% CI 0.81 to 0.98), but not for patients with tumor staged III–IV **(**HR 0.99, 95% CI 0.92 to 1.07). The *p*-value for the interaction term was 0.08. Figure 3 presents the predicted cumulative survival.

In the sensitivity analyses excluding patients who survived less than 2 months after entering the OSCAR study, the association between dispositional optimism and all-cause mortality remained almost unchanged (results not shown).

## Discussion

This Swedish nationwide population-based cohort study found that in the period before the COVID-19 pandemic, higher dispositional optimism was associated with lower all-cause mortality after esophageal cancer surgery among patients with tumor staged Tis-II. However, among those with advanced tumor stage (III–IV) and during the COVID-19 pandemic, non-significant associations were observed.

The current study adds to the growing evidence of the beneficial effect of dispositional optimism on survival [13–24]. Several potential biological, behavioral, and psychosocial pathways may explain the observed protective effect. Higher dispositional optimism has been found to be associated with healthier levels of several aging biomarkers including telomere, fibrinogen, interleukin-6, interleukin-10, homocysteine, and lipid profile [36–39], and thus, it may be related to lower risks of developing and dying from age-related diseases such as cardiovascular diseases, which is the leading non-cancer cause of death in patients with esophageal cancer in Sweden [40]. Moreover, people with higher dispositional optimism tend to be non-smokers, exercise more, and have healthier dietary habits [41]. Such healthier lifestyles may further protect them from developing comorbidities and slow the progression of already diagnosed diseases. In addition, more optimistic people are less likely to develop psychological comorbidities and feel lonely [31, 42], which may decrease the risks of mortality [8, 43]. Moreover, higher dispositional optimism predicts better health-related quality of life [44], and the latter further predicts better survival after esophageal cancer surgery [45].

The prognosis for advanced esophageal cancer is much less controllable compared with early and intermediate stages of cancer, which may explain the observed effect modification by tumor stage. It is difficult for patients with advanced esophageal cancer to prolong survival due to the aggressive tumor itself (5-year survival below 15%) [3]. However, it is increasingly possible for patients with early and intermediate esophageal cancer to achieve long-term survival (5-year survival 30–56%) [3], so they can have the chance to benefit from dispositional optimism via fewer comorbidities and slower disease progression. Future studies with larger sample sizes examining disease-specific cause of death and examining whether dispositional optimism relates to cancer recurrence might be useful to verify this speculation.

The observed predictive effect of dispositional optimism on survival may be beneficial, especially for patients with early and intermediate stages of esophageal cancer. Assessing dispositional optimism may help identify patients with a higher risk of premature death, thus leading to timely and tailored interventions. Also, given that dispositional optimism can be improved via psychological interventions like Best Possible Self exercise and cognitive behavioral therapy [11], it might be a potential intervention target to improve survival after esophageal cancer surgery.

We did not find a statistically significant association between dispositional optimism and all-cause mortality for the period within the COVID-19 pandemic. The analyzed person-time within the COVID-19 pandemic in this study was limited, which led to unstable point estimates and wide confidence intervals. Thus, this result should be interpreted with great caution and needs to be examined further by future studies using larger sample sizes. However, it makes conceptual sense that the protective effect of dispositional optimism on survival disappeared during the COVID-19 pandemic. It has been reported that individuals aged above 60 have up to 70% excess mortality during the first wave of the COVID-19 pandemic in Sweden, compared with corresponding calendar weeks in previous years [46]. Patients with esophageal cancer are one of the most vulnerable populations because of their diseases and relatively old age (mean age in the current study was 67.4 years). Moreover, lots of routine healthcare and scheduled surgeries have been postponed during the COVID-19 pandemic in Sweden [47]. Such a healthcare shortage implies that the prognosis of esophageal cancer became less controllable for all patients during the COVID-19 pandemic, which might thus nullify the protective effect of dispositional optimism. In addition, one study has found that more optimistic people tend to underestimate the severity of the COVID-19 pandemic and engage less in protective behaviors [48], such a side effect of optimism during the COVID-19 pandemic may cancel out the protective effect of optimism, thus leading to a non-significant observed association.

This study has several strengths. First, to the best of our knowledge, it is the first study examining the association between dispositional optimism and all-cause mortality after esophageal cancer surgery. Second, the comprehensive data collection allowed for adjustments for confirmed confounders as well as a selection of potential but unproven confounders, which reduced the risk of confounding bias. Third, the predefined interaction and subgroup analyses addressed the effect modifications by pathological tumor stage and the COVID-19 pandemic, and thus presented a more comprehensive picture of the association between dispositional optimism and postoperative survival. Fourth, complete follow-up based on register reduced the risk of selection bias. Lastly, the nationwide population-based prospective cohort study design enhanced the generalizability of the results.

This study also has some limitations. Because the OSCAR study focuses on patients who have survived for at least 1 year after esophagectomy, all patient-reported outcomes were started to be collected at 1 year after surgery. The results of this study might not be applicable to the period within 1 year after surgery and should be interpreted in light of the measurement time point of dispositional optimism. However, previous studies have shown that dispositional optimism keep almost unchanged before and after cancer diagnosis and surgery [34, 35]. Additionally, the analysis was conditioned on participation in the OSCAR study, which might induce selection bias, and findings of this study should be generalized cautiously to non-participants. In addition, although the sensitivity analysis excluding patients surviving less than 2 months after LOT-R measurement generated similar results as the main analysis, the possibility of reverse causality cannot be entirely ruled out as the choice of 2 months was arbitrary, albeit predefined. Lastly, although adjusting for sociodemographic variables may have partly controlled for some unmeasured confounders including genetic factors [49] and early life events [50], there is still a risk of residual confounding. Moreover, association is not equal to causation, and whether increasing dispositional optimism could improve survival needs to be further examined by future interventional research.

In conclusion, this study showed that higher dispositional optimism was associated with lower all-cause mortality after esophageal cancer surgery in patients with early and intermediate tumor stages in the period before the COVID-19 pandemic. Future studies with larger sample sizes are needed to validate the predictive effect of dispositional optimism and to identify the underlying mechanisms. Intervention studies are warranted to examine whether dispositional optimism could be a modifiable target to help improve survival after esophageal cancer surgery.

## Supplementary Information

Below is the link to the electronic supplementary material.

Supplementary file1 (PDF 159 KB)

## Data Availability

The data that support the findings of this study are stored at the server of Karolinska Institutet and are available from the corresponding author on reasonable request. The data are not publicly available due to privacy and ethical restrictions.
