# Supplementary material for: Dispositional optimism and all-cause mortality after esophageal cancer surgery: a nationwide population-based cohort study
Source: Support Care Cancer. 2022 Aug 11;30(11):9461–9. doi: 10.1007/s00520-022-07311-z (PMC9371627; doi:10.1007/s00520-022-07311-z)
Supplement: Supplementary file 1 — Supplementary file1 (PDF 159 KB) [file 520_2022_7311_MOESM1_ESM.pdf]

Supplementary Material

Article Title

Dispositional optimism and all-cause mortality after esophageal cancer surgery: a nationwide population-based cohort study

Journal Name: Supportive Care in Cancer

Authors

Yangjun Liu, PhD; Erik Pettersson, PhD; Anna Schandl, PhD; Sheraz Markar, PhD; Asif Johar, Msc; Pernilla Lagergren, PhD

Corresponding authors

Pernilla Lagergren, Department of Molecular Medicine and Surgery, Karolinska Institutet, Karolinska University Hospital, Stockholm, Sweden

Postal address: Retzius väg 13a, Level 4, 171 77 Stockholm, Sweden

E-mail: pernilla.lagergren@ki.se

Supplementary Content

Table S1. Hazard ratio (HR) and 95% confidence interval (CI) for the all-cause mortality after esophageal cancer surgery with one unit increase of the LOT-R sum score, generated by cox proportional hazards models adjusted for different covariates

Table S2. LOT-R sum score for 1-year esophageal cancer survivors with different sociodemographic and clinical characteristics

Table S1. Hazard ratio (HR) and 95% confidence interval (CI) for the all-cause mortality after esophageal cancer surgery with one unit increase of the LOT-R sum score, generated by cox proportional hazards models adjusted for different covariates

**Adjusted covariates in the model**	**HR with 95% CI**
Sociodemographic variables	0.96 (0.91, 1.02)
Predetermined variables + pathological tumor stage	0.97 (0.92, 1.02)
Predetermined variables + neoadjuvant therapy	0.96 (0.91, 1.02)
Predetermined variables + surgical approach	0.96 (0.91, 1.02)
Predetermined variables + histology	0.96 (0.91, 1.02)
Predetermined variables + postoperative complication	0.96 (0.91, 1.02)
Predetermined variables + resection margin status	0.97 (0.91, 1.02)
Predetermined variables + comorbidity	0.96 (0.91, 1.02)

Note. LOT-R: Life Orientation Test-Revised.

Table S2. LOT-R sum score for 1-year esophageal cancer survivors with different sociodemographic and clinical characteristics

	Number (%)	LOT-R sum score		
	(n = 335)	Mean	Standard deviation	*p* value
**Age**				
< 60	59 (17.6)	15.3	3.2	
60-74	216 (64.5)	15.3	3.2	0.96
≥75	60 (17.9)	15.1	3.4	
**Sex**				
Female	31 (9.3)	15.8	3.1	0.36
Male	304 (90.8)	15.2	3.3	
**Cohabitation status**				
Non-cohabitating	79 (23.6)	14.8	3.1	0.18
Cohabitating	256 (76.4)	15.4	3.3	
**Education level**				
Nine-year compulsory school	82 (24.5)	15.3	2.9	
Upper secondary school	159 (47.5)	15.2	3.5	0.97
Higher education	94 (28.1)	15.3	3.1	
**Neoadjuvant therapy**				
Yes	265 (79.1)	15.2	3.3	0.41
No	70 (20.9)	15.5	3.0	
**Surgical approach**				
Total minimally invasive esophagectomy	115 (34.3)	15.1	3.2	
Hybrid minimally invasive esophagectomy	119 (35.5)	15.4	3.5	0.87
Open esophagectomy	101 (30.2)	15.2	3.0	
**Pathological tumor stage**				
Tis–II	219 (65.4)	15.2	3.1	0.94

III–IV	116 (34.6)	15.3	3.5	
**Tumor histology**				
Adenocarcinoma	279 (83.3)	15.2	3.3	
Squamous cell carcinoma	51 (15.2)	15.2	3.3	0.97
Dysplasia	5 (1.5)	15.6	2.7	
**Postoperative complications (Clavien–Dindo grade)**				
No complication	113 (33.7)	15.1	3.3	
I–II	94 (28.1)	15.1	3.5	0.49
III–IV	128 (38.2)	15.5	3.0	
**Resection margin status**				
Radical	307 (91.6)	15.2	3.2	0.51
Nonradical	28 (8.4)	15.6	3.6	
**Charlson comorbidity index**				
0	142 (42.4)	15.3	3.3	
1	112 (33.4)	15.0	3.3	0.45
≥2	81 (24.2)	15.5	3.1	

Note. All values are number (%) unless otherwise stated, and the percentage is rounded up, which in some cases gives a sum not equal to 100%.

LOT-R: Life Orientation Test-Revised.
